# Persistence, Source Control, and Secondary Bacterial Bloodstream Infection in Catheter-Related Candidemia: A Retrospective Cohort Study

**DOI:** 10.3390/jof12080563

**Published:** 2026-08-01

**Authors:** Emel Gürcüoğlu, Gülbahar Çalışkan, Müzeyyen Tuğçe Benli, Demet Timur

**Affiliations:** 1 Department of Infectious Diseases and Clinical Microbiology, Bursa City Hospital, Bursa 16250, Türkiye; tugceninadresi@hotmail.com; 2 Department of Anesthesiology and Reanimation, Bursa City Hospital, Bursa 16250, Türkiye; alkanbahar@yahoo.com; 3 Department of Medical Microbiology, Bursa City Hospital, Bursa 16250, Türkiye

**Keywords:** source control, neutrophil-to-lymphocyte ratio, non-albicans candidemia, secondary bloodstream infection, echinocandin, EUCAST

## Abstract

Background: In catheter-related candidemia, a true catheter-source infection is often difficult to distinguish from a catheter that merely marks critical illness. Our primary aim was to identify predictors of 30-day mortality; secondary aims were to characterize species-specific patterns of persistent candidemia, its prognostic meaning within the host immune–inflammatory context, and the relationship between echinocandin therapy and secondary bacterial bloodstream infection (BSI). Methods: We retrospectively reviewed 89 consecutive episodes of catheter-related candidemia at Bursa City Hospital (2021–2025). Catheter association required *Candida* spp. growth in concurrent catheter and peripheral blood cultures. The primary endpoint was 30-day mortality; persistent candidemia (≥72 h) and secondary bacterial BSI on days 3–30 were evaluated as exploratory endpoints. Raw MIC values were retrospectively reinterpreted according to EUCAST v12.1 breakpoints (effective 10 April 2026). Results: Thirty-day mortality was 53.9% (48/89). The multivariable model was significant (LR χ^2^ = 12.12, df = 4, *p* = 0.017; Nagelkerke R^2^ = 0.170); NLR was not significantly associated with mortality (OR = 1.061, 95% CI 1.00–1.13, *p* = 0.064). Catheter removal, initial echinocandin therapy, and persistent candidemia were not independent predictors, though catheter retention was associated with higher mortality on univariable analysis (83.3%; *p* = 0.033). Persistent candidemia occurred in 51.7% of episodes, most notably with *C. parapsilosis* and *C. auris*. The NLR-persistence interaction was not formally significant, but mortality was higher when both co-occurred (66.7% vs. 36.4%; OR = 3.50, *p* = 0.075). Secondary BSI occurred in 28.4% of episodes and was more frequent in the echinocandin group (39% vs. 20%; *p* = 0.062); sensitivity analyses did not change the main findings. Conclusions: Mortality could not be explained by antifungal choice alone; these findings are exploratory. Host response, source control, species distribution, survival time, and device exposure are possible contributing factors warranting evaluation in prospective studies.

## 1. Introduction

Candidemia is the most common clinical form of invasive candidiasis, associated with high mortality in intensive care settings, and remains a major source of morbidity and mortality among hospitalized patients [1,2]. Within this population, episodes attributable to an intravascular catheter constitute a clinically distinct subgroup [3,4]. Although current guidelines recommend catheter removal [3,5], clinical practice does not always make this feasible: in patients dependent on continuous vascular access—particularly those with an implanted port or who are hemodynamically unstable—the device may not be removable immediately. How catheter management, antifungal choice, and host immune response shape the clinical course in these patients has not been adequately characterized.

Current guidelines recommend echinocandins as first-line therapy for candidemia based on efficacy and safety data [5]. In catheter-related infections, this preference has an additional mechanistic rationale: the activity of echinocandins against *Candida* biofilms within the catheter lumen offers the potential for a favorable outcome even when the catheter cannot be removed or its removal is delayed [6,7,8]. However, whether this theoretical advantage translates into a measurable clinical benefit in real-world settings—where treatment decisions are intertwined with species-specific resistance patterns, disease severity, and clinical urgency—remains uncertain.

The mechanism underlying candidemia-attributable mortality has also long been debated, and current evidence suggests that this relationship arises less from the pathogen’s virulence per se than from host- and treatment-related factors. Matched cohort studies adjusting for illness-severity scores (APACHE II, SOFA) have shown a marked reduction in attributable mortality, indicating that much of the crude mortality difference reflects the patient’s already-critical clinical condition [9]. Similarly, numerous studies have demonstrated the independent and cumulative effect of delayed initiation of appropriate antifungal therapy on mortality; failure to initiate treatment within the first 24–48 h after blood culture positivity substantially increases mortality risk [10]. Delayed source control (e.g., central venous catheter removal, abscess drainage) has likewise been associated with mortality [11]. Reported differences in mortality among *Candida* species (e.g., higher mortality associated with *C. glabrata* or *C. auris*) are largely attributable to reduced fluconazole susceptibility in these species and the resulting delay or failure of treatment, rather than a direct virulence factor. Taken together, these findings suggest that the principal determinants of candidemia-attributable mortality are the patient’s underlying clinical condition, delays in diagnosis and treatment, and the adequacy of source control, rather than the pathogen’s intrinsic virulence; however, these determinants remain inadequately characterized in the catheter-related subgroup.

What happens after the acute phase of candidemia has been relatively little studied. Patients who survive the initial episode face a second wave of risk from prolonged hospitalization, ongoing invasive devices, and secondary bacterial bloodstream infections triggered by immune dysregulation. These late-onset infections may reflect the true cost of prolonged critical illness rather than failure of the antifungal strategy, yet they are rarely examined as a distinct endpoint in candidemia studies [12].

In this study, we addressed four questions in a cohort of patients with catheter-related candidemia: What predicts 30-day mortality beyond established risk factors? Does echinocandin selection contribute to mortality and survival outcomes? How should persistent candidemia be interpreted in the context of host immune status? Does the relationship between echinocandin therapy and secondary bacterial bloodstream infection reflect a consequence of treatment choice, or does it reveal a more fundamental truth about the clinical course of these patients?

## 2. Methods

### 2.1. Study Design and Setting

This single-center retrospective cohort study was conducted at Bursa City Hospital, a 1355-bed tertiary referral center in Bursa, Türkiye. All consecutive candidemia episodes between January 2021 and December 2025 were reviewed. The study was approved by the Bursa City Hospital Scientific Research Ethics Committee (decision no. 2026-04/3, dated 18 February 2026), and the requirement for informed consent was waived owing to the retrospective design.

### 2.2. Case Definitions

Catheter-related candidemia was defined as isolation of *Candida* spp. in at least two blood culture sets from a patient with an intravascular catheter and clinical signs of infection, provided that growth was demonstrated in both catheter-drawn and concurrent or closely timed peripheral blood cultures [3,4]. Catheter association was further supported by identification of a concordant species from a catheter-tip culture and/or resolution of candidemia following catheter removal. Patients with a positive catheter-tip culture alone, without a concurrent positive blood culture, were excluded.

Persistent candidemia was defined as blood cultures remaining positive for *Candida* spp. for 72 h or longer despite antifungal therapy. Mixed *Candida*/bacterial bloodstream infection was defined as isolation of a bacterial pathogen from blood cultures drawn within 48 h before or after the index candidemia episode. For defined bacterial pathogens, a single positive blood culture accompanied by initiation or modification of antibacterial therapy was considered sufficient. For coagulase-negative staphylococci and other potential contaminants, at least two separate positive blood cultures with a concordant species and treatment decision were required.

Secondary bacterial BSI was defined as a new, microbiologically documented bacteremia episode treated with antibacterial therapy, occurring between days 3 and 30 after candidemia onset. In patients whose catheter was not removed, organisms distinct from those identified in mixed *Candida*/bacterial episodes were counted. This time window was chosen to exclude co-infections and to capture events reasonably attributable to the candidemia episode and its management.

Recatheterization was defined as placement of a new intravascular catheter following removal of the infected or suspected device. Patients were classified as having same-day recatheterization (new catheter placed within 24 h), delayed recatheterization (>24 h), or no recatheterization.

### 2.3. Microbiological Methods and Resistance Interpretation

*Candida* species identification was performed using the VITEK 2 (bioMérieux, Marcy-l’Étoile, France) and BD Phoenix (Becton, Dickinson and Company, Sparks, MD, USA) automated identification systems. Only isolates identified as *Candida auris* (also referred to as *Candidozyma auris* in current taxonomy) were confirmed by matrix-assisted laser desorption/ionization time-of-flight mass spectrometry (MALDI-TOF MS).

Antifungal susceptibility testing for *Candida* isolates other than *C. auris* was performed using the VITEK 2 (bioMérieux) automated system or the Sensititre YeastOne colorimetric broth microdilution method (Thermo Fisher Scientific, Waltham, MA, USA), depending on the method in laboratory use during the study period. Results were interpreted according to CLSI methodology and species-specific clinical breakpoints. For fluconazole, susceptible (S) ≤ 2 mg/L, susceptible dose-dependent (SDD) 4 mg/L, and resistant (R) ≥ 8 mg/L breakpoints were used for *C. albicans*, *C. parapsilosis*, and *C. tropicalis* isolates. As no susceptible category exists for *C. glabrata*, isolates were classified as SDD (≤32 mg/L) or resistant (≥64 mg/L). *C. krusei* was considered intrinsically resistant to fluconazole.

Because no CLSI clinical breakpoints have been established for this species, antifungal susceptibility results for *C. auris* isolates were interpreted using the tentative breakpoints of the United States Centers for Disease Control and Prevention (CDC) together with the current clinical breakpoints and epidemiological cutoff values (ECOFFs) of the European Committee on Antimicrobial Susceptibility Testing (EUCAST) [13,14]. As no official EUCAST clinical breakpoint exists for fluconazole, the CDC’s tentative resistance breakpoint (≥32 mg/L) was used. *C. auris* isolates with fluconazole MICs > 256 mg/L were classified as resistant.

For voriconazole, CLSI clinical breakpoints of S ≤ 0.12 mg/L, SDD 0.25–0.5 mg/L, and R ≥ 1 mg/L were applied for *C. albicans*, *C. parapsilosis*, and *C. tropicalis*; and S ≤ 0.5 mg/L, SDD 1 mg/L, and R ≥ 2 mg/L for *C. krusei*. For micafungin, breakpoints of S ≤ 0.25 mg/L, I 0.5 mg/L, and R ≥ 1 mg/L were applied for *C. albicans*, *C. tropicalis*, and *C. krusei*; S ≤ 2 mg/L, I 4 mg/L, and R ≥ 8 mg/L for *C. parapsilosis*; and S ≤ 0.06 mg/L, I 0.12 mg/L, and R ≥ 0.25 mg/L for *C. glabrata*. Because no CLSI species-specific clinical breakpoints have been established for voriconazole in *C. glabrata* or for amphotericin B in any evaluated *Candida* species, results were classified as wild-type (WT) or non-wild-type (non-WT) according to current epidemiological cutoff values. For *C. glabrata*, the epidemiological cutoff value for voriconazole was 0.25 mg/L. For amphotericin B, the epidemiological cutoff value applied throughout the study period was 2 mg/L for *C. albicans*, *C. glabrata*, *C. krusei*, and *C. tropicalis* isolates. For *C. parapsilosis*, the epidemiological cutoff value, previously 2 mg/L, was updated to 1 mg/L following the 2020 publication of the third edition of CLSI M59. Isolates with an MIC at or below the epidemiological cutoff were classified as WT, and those above it as non-WT.

### 2.4. Data Collection and Variables

Demographic, clinical, microbiological, and outcome data were obtained from electronic patient records. The neutrophil-to-lymphocyte ratio (NLR) and systemic immune–inflammation index (SII; neutrophil count × platelet count/lymphocyte count) were calculated from complete blood counts obtained on the day of index culture collection and within the following 24 h. Pre-index catheter dwell time was defined as the interval from catheter placement to candidemia onset. The Charlson Comorbidity Index (CCI) was calculated for each patient [15].

### 2.5. Endpoints

The primary endpoint was 30-day all-cause mortality following candidemia onset. Secondary endpoints included persistent candidemia, secondary bacterial BSI (days 3–30), and metastatic complications (endocarditis, endophthalmitis, neurocandidiasis, or other metastatic foci). The interaction between NLR and persistence was evaluated as an exploratory analysis.

### 2.6. Statistical Analysis

Continuous variables were expressed as median and interquartile range (IQR) and compared using the Mann–Whitney U test. Discrete/ordinal scores such as qSOFA and the Charlson Comorbidity Index (CCI) were also compared using this rank-based, distribution-free nonparametric test; the Mann–Whitney U test is an appropriate and commonly used method for ordinal data. Categorical variables were compared using Fisher’s exact test or the chi-square test, as appropriate. Multivariable logistic regression was used to assess variables associated with 30-day mortality; NLR, catheter removal, initial echinocandin therapy, and persistent candidemia were entered into the model, with NLR modeled as a continuous variable. To assess the robustness of the primary model, sensitivity analyses were performed by adding concurrent bacteremia (±48 h) and secondary bacterial BSI (days 3–30) separately (not simultaneously in the same model) as a fifth covariate, in accordance with the 10-events-per-variable rule; the original model was also repeated in the “pure” candidemia subgroup without secondary BSI (*n* = 63). A two-sided *p*-value < 0.05 was considered statistically significant. Given the limited number of outcome events (~48 deaths), multivariable models were restricted to a maximum of 4–5 covariates in accordance with the 10-events-per-variable rule.

## 3. Results

### 3.1. Study Population

**Figure 1:**
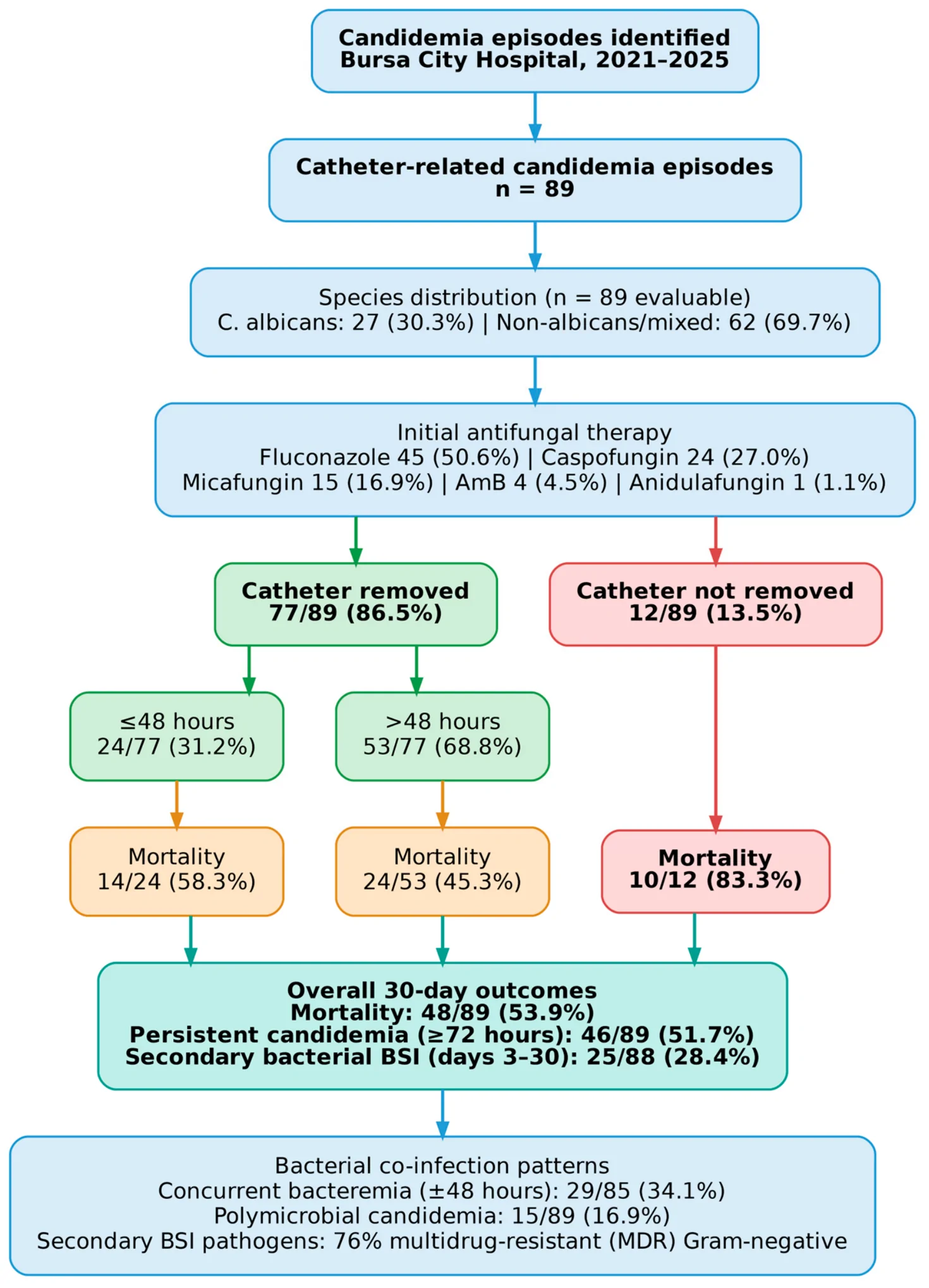
Cohort flow, initial antifungal therapy, catheter management, and 30-day outcomes. Flow diagram showing species distribution, initial antifungal therapy, timing of catheter removal, bacterial co-infection patterns, and 30-day outcomes among 89 catheter-related candidemia episodes treated at Bursa City Hospital between 2021 and 2025. Species distribution was evaluable in all 89 episodes; *C. albicans* was identified in 27 episodes (30.3%), and non-albicans or mixed-species episodes in 62 (69.7%).

**Figure 2:**
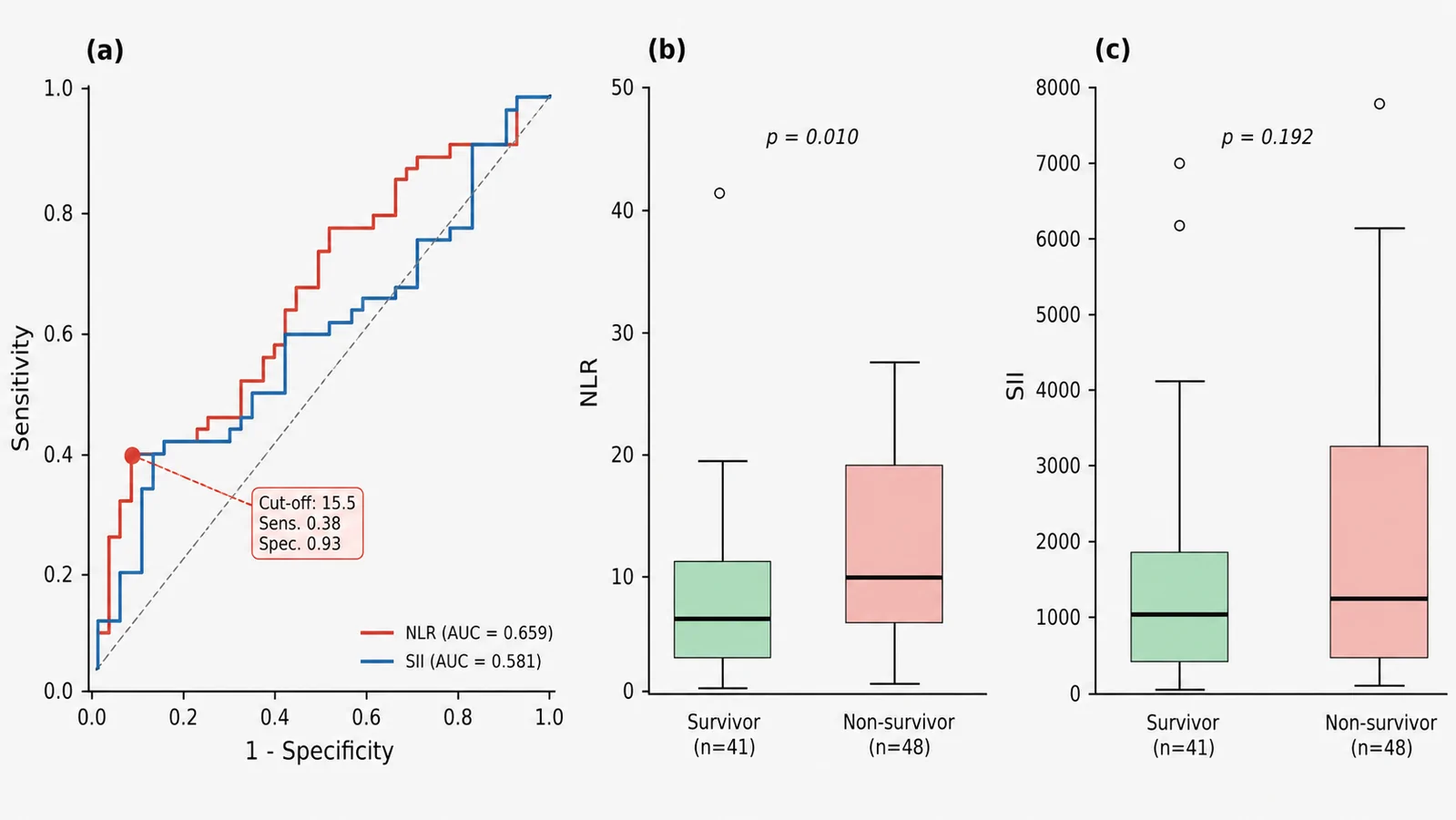
Discriminative Performance of NLR and SII for 30-day mortality. (**a**,**b**) Distribution of NLR and SII among survivors and non-survivors, respectively. Circles represent individual patient observations, and horizontal lines indicate the median values. (**c**) ROC curves for NLR and SII. NLR showed modest discrimination (AUC = 0.659). The optimal NLR cut-off was 15.5, with a sensitivity of 0.38 and a specificity of 0.93.

A total of 89 catheter-related candidemia episodes were included in the study (Table 1; Figure 1). The median age was 68 years (IQR 57–77), with a slight female predominance (56.2%). Approximately three-quarters of patients (74.2%) were in the intensive care unit (ICU) at candidemia onset. Regarding the clinical unit in which patients were located at the time of the index culture, 54 patients (60.7%) were in the ICU, 17 (19.1%) in internal medicine, 9 (10.1%) in general surgery, and 9 (10.1%) in the burn unit. The median Charlson Comorbidity Index was 5 (IQR 3–7), reflecting a substantial comorbidity burden. Mechanical ventilation was present in 47.2% of patients at the time of the index culture. The median pre-index catheter dwell time was 19 days (IQR 10–33). NLR and SII findings in relation to 30-day mortality are presented in Figure 2.

### 3.2. Species Distribution and Antifungal Resistance

Species distribution and resistance profiles are presented in Table 2. *C. albicans* was isolated in 27 patients (30.3%), followed by episodes involving *C. parapsilosis* in 34 patients (38.2%), *C. tropicalis* in 8 (9.0%), *C. auris* in 7 (7.9%), and *C. glabrata* in 12 (13.5%). Polymicrobial candidemia (two or more *Candida* species) occurred in 15 episodes (16.9%).

Azole resistance, interpreted according to EUCAST v12.1 clinical breakpoints, showed species-specific patterns. All *C. albicans* isolates (27/27) were fluconazole-susceptible. Of 29 *C. parapsilosis* isolates, 24 (82.8%) showed fluconazole resistance (MIC 16 mg/L, exceeding the EUCAST R > 4 mg/L breakpoint). All tested *C. auris* isolates showed very high fluconazole MICs, exceeding 256 mg/L; however, because EUCAST has not defined an official clinical breakpoint or ECOFF for this species-drug combination, these isolates were not categorically classified as resistant (R) and were reported only by their observed MIC values. Updated EUCAST breakpoints published in early 2026 now allow formal clinical assessment for amphotericin B and echinocandins [14]. *C. glabrata* isolates were classified as “I” (susceptible, increased exposure) according to the current EUCAST Clinical Breakpoint Table, as EUCAST does not define a separate S/R breakpoint for this species–drug combination; clinically, these isolates were managed as having reduced azole susceptibility. *C. tropicalis* isolates were fully susceptible. All isolates tested were susceptible to amphotericin B, anidulafungin, and micafungin in addition to fluconazole; as EUCAST has not established an official breakpoint for caspofungin, caspofungin susceptibility was inferred indirectly from anidulafungin and micafungin results. No resistant isolates were detected in any of the combinations tested. The overall azole non-susceptibility rate (restricted to fluconazole-species combinations with an official R breakpoint) was 34.8% (31/89), driven mainly by *C. parapsilosis*; *C. auris* isolates were not included in this rate, as no official EUCAST fluconazole breakpoint exists for this species. *C. glabrata* isolates were assessed within the “I” (susceptible, increased exposure) category defined by EUCAST for the entire wild-type population, reflecting reduced susceptibility without being equated with categorical resistance.

### 3.3. Antifungal Therapy and Catheter Management

**Figure 3:**
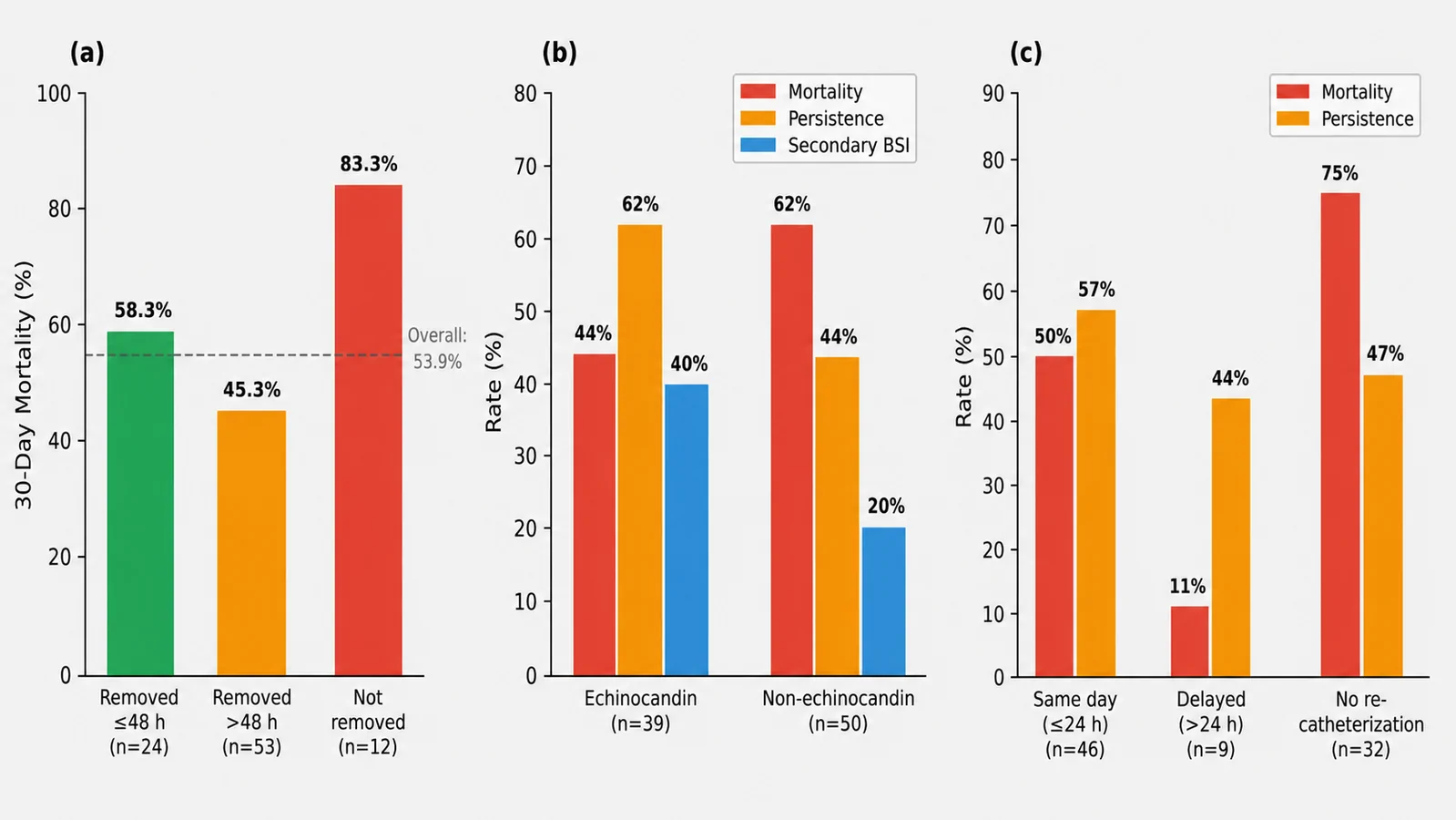
Clinical outcomes by catheter management, antifungal strategy, and recatheterization timing. (**a**) Thirty-day mortality by timing of catheter removal. (**b**) Mortality, persistent candidemia, and secondary BSI by initial antifungal strategy. (**c**) Mortality and persistent candidemia by recatheterization timing. These subgroup analyses are exploratory and should not be interpreted as evidence of causality.

Initial antifungal therapy was fluconazole in 45 patients (50.6%), caspofungin in 24 (27.0%), micafungin in 15 (16.9%), amphotericin B in 4 (4.5%), and anidulafungin in 1 (1.1%) (Table 3; Figure 3b). Echinocandins were initiated in 39 patients (43.8%). Critically, antifungal class selection was strongly determined by species: none of the 27 *C. albicans* patients received an echinocandin as initial therapy, whereas all 7 *C. auris* patients did. Early antifungal initiation (≤24 h) occurred in 31.5% of episodes [10].

Catheters were removed in 77 patients (86.5%) and left in place in 12 (13.5%) (Figure 3a). Of the 12 patients whose catheter was left in place, 9 had an implanted port that could not be removed owing to clinical constraints, and mortality in this subgroup was 83.3% (10/12). Catheter removal within 48 h of candidemia onset occurred in 24 patients. Same-day recatheterization occurred in 46 patients (51.7%), delayed recatheterization (>24 h) in 9, and no recatheterization in 32 (Table 4; Figure 3c).

### 3.4. Mortality and Its Determinants

Overall 30-day mortality was 53.9% (48/89) (Table 3). On univariable analysis, NLR was the only continuous inflammatory marker significantly associated with mortality (median 9.6 among non-survivors vs. 6.1 among survivors; *p* = 0.010). SII showed an effect in the same direction but did not reach statistical significance (*p* = 0.192); age, CCI, lactate, and qSOFA did not differ significantly between survivors and non-survivors. Catheter retention was significantly associated with mortality (*p* = 0.033). ROC analysis showed moderate discrimination for NLR (AUC = 0.659) and lower discrimination for SII (AUC = 0.581); the optimal NLR cut-off was 15.5, with a sensitivity of 0.38 and a specificity of 0.93 (Figure 2).

In the multivariable logistic regression model for 30-day mortality, the overall model was statistically significant (likelihood ratio χ^2^ = 12.12, df = 4, *p* = 0.017; Nagelkerke R^2^ = 0.170). NLR showed a statistically non-significant association with mortality (OR per unit increase 1.061, 95% CI 1.00–1.13, *p* = 0.064); catheter removal, initial echinocandin therapy, and persistent candidemia were not independently associated with 30-day mortality in this model (Table 5). A Nagelkerke R^2^ of 0.170 indicates that the model explains approximately 17% of the variance in 30-day mortality, reflecting limited predictive power.

To assess the robustness of the model, two sensitivity analyses were performed by separately adding concurrent bacteremia (±48 h) and secondary bacterial BSI (days 3–30) as a fifth covariate (see Section 2.6). When concurrent bacteremia was added (*n* = 85; four patients had missing data for this variable), this variable was not independently associated with mortality (OR 0.755, 95% CI 0.29–1.95, *p* = 0.561); the effect size of NLR remained similar (OR 1.054, *p* = 0.097), and the explanatory power of the model decreased slightly (Nagelkerke R^2^ = 0.159; overall model *p* = 0.051). When secondary bacterial BSI was added (*n* = 88; one patient had missing data for this variable), this variable was also not independently associated with mortality (OR 0.506, 95% CI 0.18–1.39, *p* = 0.186); its direction, below 1 (apparently protective), is consistent with a possible survivor bias arising from the fact that secondary BSI can only be observed in patients who survive long enough (see Section 3.6 and Section 4) and should not be interpreted as a protective effect. In this model, Nagelkerke R^2^ increased to 0.202 (overall model *p* = 0.013). In both sensitivity analyses, the direction and significance of the effects of NLR, catheter removal, initial echinocandin therapy, and persistent candidemia were consistent with the original four-variable model; that is, adjusting for concurrent bacterial infections did not change the main findings. In addition, the original four-variable model was repeated in the “pure” candidemia subgroup without secondary bacterial BSI (*n* = 63; 30-day mortality in this subgroup was 60.3%); the direction and magnitude of the NLR effect were preserved (OR 1.060, 95% CI 0.99–1.14, *p* = 0.106), although the overall model did not reach statistical significance owing to the smaller sample size (*p* = 0.094; Nagelkerke R^2^ = 0.160). These findings suggest that the direction of the main associations was consistent across subgroups, but that the sample size was insufficient for confirmatory inference.

Temporal analysis of mortality revealed distinct patterns. Six patients (6.7%) died within 7 days of candidemia onset; none had received echinocandin therapy, and half had concurrent bacterial BSI. This subgroup had the highest NLR values and represented patients in whom disease severity outpaced any treatment intervention. The largest mortality subgroup (27 patients, 30.3%) died after day 14; this group was characterized by persistent candidemia (59%), mechanical ventilation (59%), and development of secondary bacterial BSI (26%).

### 3.5. Persistent Candidemia

Persistent candidemia (≥72 h) occurred in 46 patients (51.7%). Species distribution within the persistent group was dominated by *C. parapsilosis* (35%), *C. albicans* (24%), and *C. auris* (9%). Metastatic complications, including two cases of endocarditis and one of endophthalmitis, were documented in 3 of 46 persistent cases (6.5%); however, this is likely an underestimate, as systematic echocardiographic and ophthalmologic screening was not performed.

Persistent candidemia (≥72 h) alone did not appear to explain 30-day mortality; mortality rates were similar in non-persistent and persistent cases (55.8% vs. 52.2%).

### 3.6. Secondary Bacterial BSI: Patterns and Risk Factors

**Figure 4:**
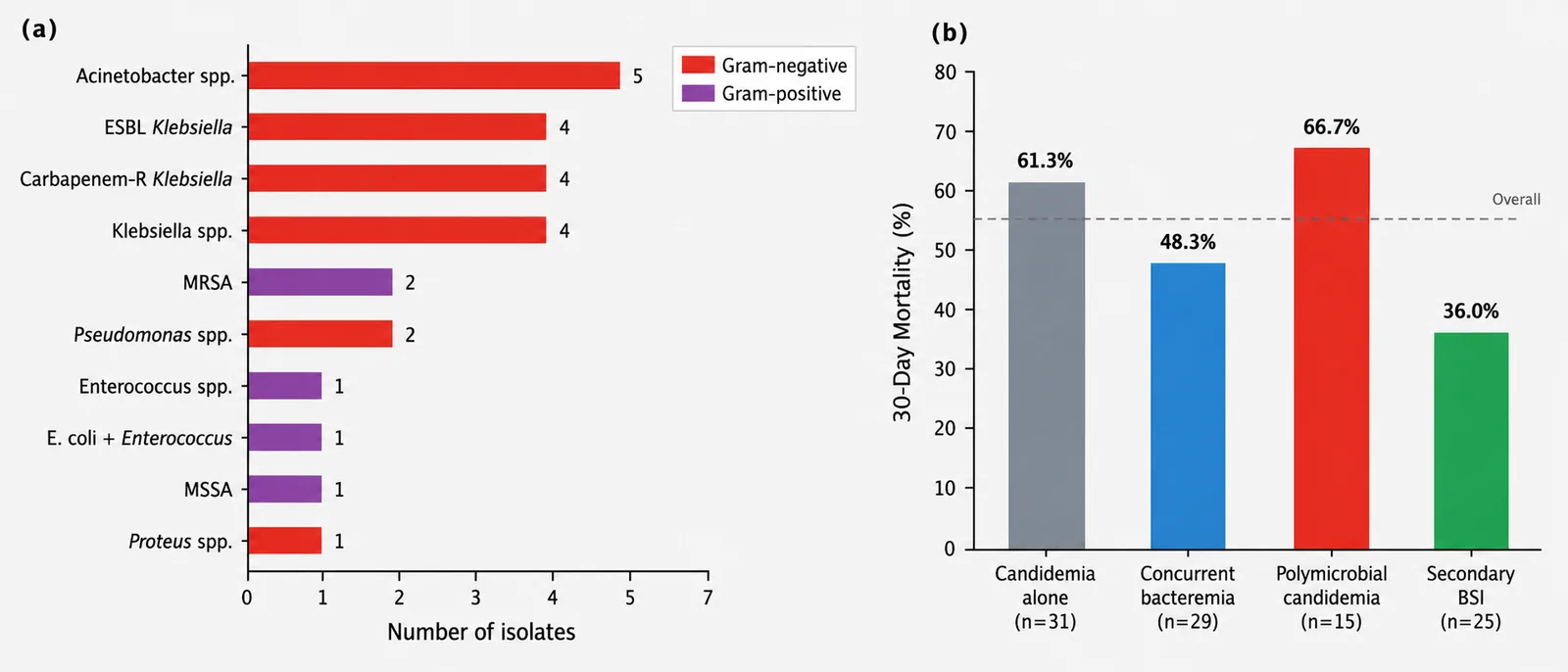
Secondary bloodstream infections and mortality by co-infection pattern. (**a**) Pathogen distribution among secondary bacterial BSIs. (**b**) Thirty-day mortality by candidemia and bacterial co-infection pattern. The lower mortality observed in secondary BSI cases should be interpreted cautiously, as secondary BSI can only be observed in patients who survive long enough to develop it, raising the possibility of survivor bias.

Secondary bacterial BSI developed in 25 patients (28.4%) between days 3 and 30 (Figure 4a). The predominant pathogens were *Acinetobacter* spp. (*n* = 5), ESBL-producing *Klebsiella* (*n* = 4), carbapenem-resistant *Klebsiella* (*n* = 4), *Klebsiella* spp. (*n* = 4), MRSA (*n* = 2), and *Pseudomonas* spp. (*n* = 2). The markedly Gram-negative, multidrug-resistant profile of these secondary infections points strongly to nosocomial acquisition during prolonged ICU stay and ongoing invasive device use, rather than a consequence of antifungal choice. Mortality by candidemia and co-infection pattern is summarized in Figure 4b.

Secondary BSI was more frequent in patients treated with echinocandins (15/38, 39%) than in those who did not receive an echinocandin (10/50, 20%), with a trend approaching significance (OR 2.50, *p* = 0.062). However, this association reflects several layers of confounding. First, patients treated with echinocandins had a higher rate of mechanical ventilation (62% vs. 36%), and all *C. auris* cases were in this group. Second, and more fundamentally, capturing a secondary BSI requires survival beyond day 3. Early mortality was higher in the non-echinocandin group, meaning fewer patients remained at risk for late-onset complications. This is a classic competing-risk scenario: early death precludes the development of secondary BSI.

Same-day recatheterization was performed in 46 patients (51.7%) and this subgroup showed the highest rate of secondary BSI compared with patients who did not undergo recatheterization (33% vs. 19%) (Table 4). Delayed recatheterization (>24 h) was associated with a numerically lower 30-day mortality compared with same-day recatheterization (11.1% vs. 50.0%; OR 0.12, Fisher’s exact *p* = 0.062), but this finding should be interpreted as exploratory given the small delayed-recatheterization subgroup (Table 6).

Among the 15 echinocandin-treated patients who developed secondary BSI, most had undergone same-day recatheterization, and 9 of 15 (60%) had persistent candidemia. Secondary pathogens were predominantly healthcare-associated Gram-negative organisms (Figure 4a), consistent with nosocomial acquisition related to critical illness and ongoing device exposure rather than direct antifungal toxicity.

### 3.7. Treatment Concordance and Outcomes

When patients were stratified by concordance between azole susceptibility and initial antifungal choice, an instructive pattern emerged. Patients with azole-resistant isolates who received concordant echinocandin therapy had the lowest mortality (40%, 12/30). Patients with azole-susceptible isolates treated with fluconazole—generally considered appropriate therapy—had the highest mortality (63%, 27/43). This paradox is explained by underlying severity profiles: azole-susceptible patients were predominantly *C. albicans* cases in the most critically ill hosts, and their higher mortality reflects the overall clinical severity of the cohort rather than treatment inadequacy. The clinical lesson is that species-directed therapy guided by identification and susceptibility data outperforms empirical coverage—not because the drug itself differed, but because the clinical attention paid to microbiological results signals a more individualized treatment approach.

### 3.8. Catheter Type and Port-Related Outcomes

Catheter type significantly influenced management options and outcomes. Among patients with a CVC (*n* = 52; three patients with unrecorded catheter type were classified as CVC following clinical data review), mortality was 46.2% (24/52) and the catheter removal rate was 98.1% (51/52). Among patients with an FVC (*n* = 27), mortality was 59% and the removal rate was 93%. Patients with a port (*n* = 10) had the highest mortality (80%) and the lowest removal rate (10%). In nine of ten port patients, the device was left in place owing to clinical necessity; seven of these nine patients died. The port subgroup showed a disproportionately high representation of infections involving *C. glabrata* (40%), illustrating the clinical dilemma of source control in patients dependent on long-term vascular access.

## 4. Discussion

Mortality in candidemia remains high, particularly in episodes that are ICU-acquired, catheter-related, persistent, or associated with septic shock [1,2,11,12,16,17]. In our cohort, the clinical course appears to have been shaped by the combined effects of host inflammatory response, whether the catheter could actually be removed, *Candida* species distribution, time to candidemia clearance, and subsequent secondary bacterial infections. In this context, catheter removal, initial echinocandin therapy, and persistent candidemia were not independent predictors of 30-day mortality.

The most consistent signal came from NLR at candidemia onset. Although its adjusted association did not reach statistical significance, NLR may still be clinically informative because it captures two opposing components of the host response: neutrophilia as a marker of acute inflammatory burden, and lymphopenia as a marker of immune exhaustion or diminished host defense. Prognosis in candidemia should therefore be read not only through fungal burden, but also through the host response to that burden. This may explain why NLR carries information that lactate or qSOFA do not fully capture [18]. No single, universally accepted NLR cut-off exists in the literature for sepsis/septic shock; published thresholds vary widely, from approximately 9 to 23, across studies [19,20], and the value of 15.5 observed in this study falls within this range and is close to values reported in time-dependent NLR studies (15.48 on day 3, 15.85 on day 5). This heterogeneity indicates that adopting an externally “validated” threshold may be at least as arbitrary as a threshold derived from our own sample; NLR was therefore modeled as a continuous variable throughout the study, without any dichotomization (threshold-based binary grouping).

Persistent candidemia alone did not explain mortality; mortality rates were similar in persistent and non-persistent cases. What may matter more is the clinical and host-immune context in which persistence occurs: in a patient with a low inflammatory response, persistently positive cultures—particularly with biofilm-forming species such as *C. parapsilosis*—may reflect ongoing seeding from the catheter surface and an unresolved source-control problem [6,7,8,12]; in contrast, persistence accompanied by a more pronounced inflammatory response may be more concerning for impaired host control, progressive infection, or critical illness. This distinction was not directly tested statistically in this study, and because biofilm burden was not directly measured, it should be regarded not as mechanistic evidence but as a clinical hypothesis that could be tested prospectively in the future.

Species distribution also warrants attention. Non-albicans or mixed *Candida* episodes accounted for 69.7% of cases, with *C. parapsilosis* the most common single species. This pattern is consistent with European ICU, Brazilian multicenter candidemia, and long-term antifungal surveillance data [1,2,21]. *C. auris* accounted for 7.9% of episodes. All tested *C. auris* isolates showed high-level azole resistance, all *C. auris* patients received echinocandin therapy from the outset, and persistent candidemia developed in four of seven cases. This co-occurrence of resistance and persistence illustrates why *C. auris* remains difficult to manage. Its clustering in the ICU also raises a transmission question beyond the scope of this study, but supports the need for enhanced infection-control surveillance [22,23,24].

The azole resistance findings argue against the assumption that empirical fluconazole remains appropriate across all non-albicans cases. Although *C. parapsilosis* is generally considered azole-susceptible, the 16 mg/L MIC values in our isolates fall within the resistant range according to EUCAST criteria [25,26]. For *C. glabrata*, EUCAST’s classification of the species in the “I” (susceptible, increased exposure) category, rather than a separate S/R breakpoint for fluconazole, makes an echinocandin-based approach more rational [5,9]. High-level fluconazole resistance in *C. auris* was also consistent with current international data [24]. Antifungal selection in this cohort should therefore be interpreted not as a simple fluconazole-versus-echinocandin comparison, but together with species identification and susceptibility results.

Secondary bacterial BSI was more frequent among patients treated with echinocandins (39% vs. 20%; *p* = 0.062). This difference may be read as a harmful effect of echinocandins; however, the findings point to a different explanation. Patients receiving echinocandins were more severely ill at baseline: mechanical ventilation was more common, all *C. auris* cases were in this group, and ICU exposure was greater. Secondary BSI also requires time at risk. Patients who died early could not survive long enough to develop late bacteremia. The excess of secondary BSI in the echinocandin group is therefore more consistent with confounding by indication, longer survival, and prolonged device exposure than with treatment-related harm. The microbiology supports this interpretation: *Acinetobacter*, carbapenem-resistant *Klebsiella*, *Pseudomonas*, and ESBL-producing organisms are more consistent with complications of prolonged ICU stay and intravascular device use than with an antifungal-related effect [27].

Recatheterization is closely related to this issue. In more than half of patients, a new catheter was placed on the same day as removal of the infected catheter, and this group had the highest rate of secondary BSI (33%) [12,28]. Delayed recatheterization was associated with a numerically lower 30-day mortality compared with same-day recatheterization (11.1% vs. 50.0%), but the delayed group comprised only nine patients; this finding should remain exploratory (*p* = 0.062). This creates a practical clinical dilemma. Removal of the infected catheter is necessary for source control, but immediate placement of a new catheter may create another portal of entry for nosocomial bacteria in a critically ill host. Whether a brief catheter-free interval could reduce the risk of secondary BSI is an important question raised, but not conclusively answered, by this study.

Port-related candidemia represents the subgroup in which source control is most difficult. Mortality was 80%, while the catheter removal rate was only 10%. Port removal is often clinically difficult in patients with cancer, a requirement for total parenteral nutrition, or a need for long-term vascular access. The higher frequency of infections involving *C. glabrata* in this group added further complexity due to reduced azole susceptibility. The high mortality among port patients should therefore not be attributed to catheter type alone; rather, it reflects the combined burden of difficult source control, host frailty, and resistant species [5,24].

The treatment concordance analysis reinforces this point. Mortality was lower among patients with azole-resistant isolates who received concordant echinocandin therapy (40%), while mortality was higher among patients with azole-susceptible isolates treated with fluconazole (63%). However, this difference does not mean fluconazole failed: the azole-susceptible/fluconazole group consisted mainly of clinically severe *C. albicans* cases. In contrast, echinocandin use in resistant species reflected a more targeted approach guided by species identification and susceptibility data [29]. The relevant issue is not simply which drug was used, but whether treatment was adjusted in a timely manner according to microbiological evidence.

Metastatic complications were documented in only 6% of cases. This rate may be underestimated, as systematic echocardiography and fundoscopy were not performed. Nevertheless, endocarditis and endophthalmitis occurred only in the persistent-candidemia group. This finding supports careful evaluation for metastatic foci in patients with persistent candidemia and continuation of antifungal therapy for at least 14 days after blood culture clearance [5].

### Limitations

This study has several limitations. Its retrospective, single-center design limits generalizability and leaves room for unmeasured confounding. The sample size of 89 patients was modest, and the statistical power of subgroup analyses was limited, increasing the risk of Type II error. For example, although 30-day mortality in the echinocandin group (43.6%) suggested a clinically meaningful difference compared with the non-echinocandin group (62.0%), this difference did not reach statistical significance owing to insufficient sample size (*p* = 0.092). The Nagelkerke R^2^ of the multivariable model was 0.170, indicating that the model explains only approximately 17% of the variance in 30-day mortality and therefore has limited predictive power; as such, the model should be regarded as hypothesis-generating rather than suitable for clinical decision support. Accordingly, the statistically non-significant multivariable association of NLR, the non-significant NLR-persistence interaction, and the recatheterization signal should be interpreted as hypothesis-generating rather than confirmatory. Sensitivity analyses adding concurrent bacteremia and secondary bacterial BSI separately to the model did not change the main findings, but findings related to secondary BSI should be interpreted cautiously owing to possible survivor bias, as no formal competing-risk analysis was performed for this variable. In the dataset, secondary BSI was recorded only as a binary (yes/no) variable, without the specific day of the event, so a Cox proportional hazards model with a time-dependent covariate could not be constructed with the available data. *Candida* species and antifungal class selection were closely related, and this confounding cannot be fully resolved without prospective randomization. Because systematic echocardiography and fundoscopy were not performed, metastatic complications may have been underdetected. SOFA scores and complete culture-clearance times were not consistently available for all patients. Although the clinical unit in which patients were located at the time of the index culture (ICU/internal medicine/surgery/burn unit) was recorded, the specific clinical indication underlying ICU or hospital admission (e.g., sepsis, respiratory failure, post-surgical monitoring, trauma) was not systematically recorded as a structured variable in this retrospective dataset; this limits full interpretation of baseline disease severity and the rationale for ICU admission. In addition, antifungal susceptibility categories (particularly for caspofungin and *C. auris*–amphotericin B) were updated in a second technical review according to EUCAST v12.1 methodological rules; caspofungin susceptibility was inferred indirectly from anidulafungin/micafungin results owing to the absence of an official EUCAST breakpoint, and susceptibility of *C. auris* isolates to amphotericin B is presented cautiously owing to EUCAST’s unusually low S breakpoint (≤0.001 mg/L). Confirmation of the raw MIC values for these isolates from laboratory records is required for definitive categorical classification and was beyond the scope of this study.

## 5. Conclusions

In catheter-related candidemia, 30-day mortality was not explained by antifungal class alone. The clinical course was shaped by host inflammatory response, catheter removability, species distribution, candidemia persistence, and subsequent device-related bacterial complications. NLR at candidemia onset provided the most notable inflammatory signal in this cohort; however, its statistically non-significant association on multivariable analysis means it should be regarded as a candidate prognostic marker requiring prospective validation. Persistent candidemia likewise requires careful interpretation. In this study, persistence alone did not increase mortality; its clinical meaning appeared to vary according to the host’s inflammatory profile. Persistence accompanied by low NLR may reflect ongoing catheter-surface seeding and difficulty with source control, whereas persistence accompanied by high NLR may indicate impaired host control or progressive critical illness. The higher rate of secondary bacterial BSI among echinocandin-treated patients should not be interpreted as direct antifungal harm. These patients were more severely ill clinically, included all *C. auris* cases, survived long enough for late nosocomial events to develop, and frequently required ongoing vascular access. Taken together, and bearing in mind the limited sample size and explanatory power of the model (Nagelkerke R^2^ = 0.170), these findings may support a management approach that moves beyond antifungal choice alone to integrate immune–inflammatory status, species-level resistance, feasibility of source control, and recatheterization timing; these hypotheses require confirmation in prospective studies.

## Figures and Tables

**Table 1:** Baseline characteristics and univariable analysis for 30-day mortality.

Variable	Total (*n* = 89)	Survivors (*n* = 41)	Non-Survivors (*n* = 48)	*p*
**Demographic characteristics**				
Age, years	68.0 (57.0–77.0)	67.0 (60.0–77.0)	69.0 (56.0–77.0)	0.767
Male sex	39/89 (43.8%)	22/41 (53.7%)	17/48 (35.4%)	0.092
CCI	5.0 (3.0–7.0)	5.0 (3.0–7.0)	5.0 (2.8–7.0)	0.864
**Severity at index**				
ICU admission	66/89 (74.2%)	28/41 (68.3%)	38/48 (79.2%)	0.332
Mechanical ventilation	42/89 (47.2%)	17/41 (41.5%)	25/48 (52.1%)	0.395
qSOFA score	1.0 (0.0–2.0)	1.0 (0.0–1.0)	1.0 (0.0–2.0)	0.344
Lactate, mmol/L	1.9 (0.9–3.4)	1.6 (0.9–2.5)	2.1 (1.1–3.4)	0.153
NLR	8.1 (4.1–13.2)	6.1 (2.9–10.8)	9.6 (5.7–18.3)	0.010 *
SII	1136.1 (404.0–2496.9)	1014.4 (404.0–1867.1)	1246.8 (471.3–3145.0)	0.192
**Comorbidities**				
Diabetes mellitus	42/89 (47.2%)	20/41 (48.8%)	22/48 (45.8%)	0.833
TPN	53/89 (59.6%)	24/41 (58.5%)	29/48 (60.4%)	1.000
Immunosuppression	22/89 (24.7%)	8/41 (19.5%)	14/48 (29.2%)	0.333
**Catheter management**				
Pre-index dwell time, days	19.0 (10.0–33.0)	19.0 (7.0–33.0)	23.5 (11.0–35.2)	0.521
Catheter removed	77/89 (86.5%)	39/41 (95.1%)	38/48 (79.2%)	0.033 *
Removal within 48 h	24/77 (31.2%)	10/39 (25.6%)	14/38 (36.8%)	0.332
**Treatment**				
Initial echinocandin	39/89 (43.8%)	22/41 (53.7%)	17/48 (35.4%)	0.092
Early antifungal (≤24 h)	28/89 (31.5%)	10/41 (24.4%)	18/48 (37.5%)	0.253
**Microbiology**				
Non-albicans/mixed	62/89 (69.7%)	31/41 (75.6%)	31/48 (64.6%)	0.355
Persistent candidemia	46/89 (51.7%)	22/41 (53.7%)	24/48 (50.0%)	0.832
Polymicrobial candidemia	15/89 (16.9%)	5/41 (12.2%)	10/48 (20.8%)	0.396
Concurrent bacteremia ± 48 h	29/85 (34.1%)	15/39 (38.5%)	14/46 (30.4%)	0.502
Secondary BSI (days 3–30)	25/88 (28.4%)	16/41 (39.0%)	9/47 (19.1%)	0.057

Continuous variables: median (IQR). Categorical variables: n/N (%). *p*-values: Mann–Whitney U test for continuous variables, Fisher’s exact test for categorical variables. CCI, Charlson Comorbidity Index; NLR, neutrophil-to-lymphocyte ratio; SII, systemic immune–inflammation index; BSI, bloodstream infection. * *p* < 0.05.

**Table 2:** Candida species distribution, antifungal susceptibility, and clinical outcomes.

Species	*n*	S	R	NT	30-d Mort. (%)	Persist. (%)	EUCAST BP
*C. parapsilosis*	29	5	24	0	34	66	S ≤ 2, R > 4
*C. albicans*	27	27	0	0	63	41	S ≤ 2, R > 4
*C. tropicalis*	8	8	0	0	62	38	S ≤ 2, R > 4
*C. glabrata + C. albicans*	7	5	2	0	57	43	I
*C. auris*	7	0	6 b	1	57	57	No BP (MIC > 256)
*C. albicans + C. parapsilosis*	3	—	—	—	67	33	—
*C. glabrata* + other a	3	0	3	0	67	33	I
*C. kefyr + C. albicans*	1	1	0	0	100	100	—
*C. tropicalis + C. parapsilosis*	1	—	—	—	100	100	—
*C. tropicalis + C. glabrata*	1	—	—	—	100	0	I
*C. parapsilosis + C. krusei*	1	0	0	1	0	100	—
*C. glabrata + C. lipolytica*	1	—	—	—	100	100	I

Azole susceptibility interpreted according to EUCAST v12.1 clinical breakpoints (effective from 10 April 2026). S, susceptible; R, resistant; NT, not tested; BP, breakpoint. Because no resistant isolates were detected for amphotericin B, anidulafungin, micafungin, voriconazole, or posaconazole among the isolates tested, detailed S/R distributions for these agents are not shown separately; caspofungin susceptibility was inferred indirectly from anidulafungin/micafungin results, as EUCAST has not established an official breakpoint for caspofungin. Only fluconazole results, for which species-specific resistance differences were observed, are presented. a Species not further specified. b EUCAST has not established an official clinical breakpoint for fluconazole against C. auris; although these isolates showed MICs > 256 mg/L, they should be classified as breakpoint not established (No BP) rather than categorically R.

**Table 3:** Clinical outcomes by initial antifungal class.

Outcome	Total (*n* = 89)	Echinocandin (*n* = 39)	Non-Echinocandin (*n* = 50)	*p*
30-day mortality	48/89 (53.9%)	17/39 (43.6%)	31/50 (62.0%)	0.092
Persistent candidemia	46/89 (51.7%)	24/39 (61.5%)	22/50 (44.0%)	0.135
Secondary BSI (days 3–30)	25/88 (28.4%)	15/38 (39.5%)	10/50 (20.0%)	0.062
Polymicrobial candidemia	15/89 (16.9%)	7/39 (17.9%)	8/50 (16.0%)	1.000
Concurrent bacteremia ± 48 h	29/85 (34.1%)	13/37 (35.1%)	16/48 (33.3%)	1.000

Fisher’s exact test. Echinocandin selection was strongly associated with species distribution. Direct comparison should be interpreted cautiously owing to indication bias.

**Table 4:** Timing of recatheterization and clinical outcomes.

Recatheterization	*n*	30-Day Mortality	Persistent Candidemia	Secondary BSI (days 3–30)
Same-day (≤24 h)	46	23/46 (50.0%)	26/46 (56.5%)	15/46 (32.6%)
Delayed (>24 h)	9	1/9 (11.1%)	4/9 (44.4%)	3/9 (33.3%)
No recatheterization	32	24/32 (75.0%)	15/32 (46.9%)	6/32 (18.8%)

Same-day recatheterization: new catheter placed within 24 h of removal of the infected catheter. Two patients with unknown status were excluded.

**Table 5:** Multivariable logistic regression model for 30-day mortality.

Variable	Odds Ratio	95% CI	*p*-Value
NLR, continuous	1.061	1.00–1.13	0.064
Catheter removal	0.345	0.06–1.93	0.226
Initial echinocandin therapy	0.613	0.24–1.54	0.299
Persistent candidemia	0.931	0.37–2.33	0.879

Model: LR χ^2^(4) = 12.12, *p* = 0.017; Nagelkerke R^2^ = 0.170.

**Table 6:** Timing of recatheterization and 30-day mortality.

Recatheterization Timing	*n*	30-Day Mortality	Comparison	OR; *p*-Value
Same-day (≤24 h)	46	50.0%	Reference	—
Delayed (>24 h)	9	11.1%	Delayed vs. same-day	OR 0.12; *p* = 0.062
No recatheterization	32	75.0%	Descriptive subgroup	Not tested

Fisher’s exact test. Exploratory; limited by the small delayed subgroup.

## Data Availability

The data presented in this study are available on reasonable request from the corresponding author.

## References

[1] Bassetti M., Giacobbe D.R., Vena A., Trucchi C., Ansaldi F., Antonelli M., Adamkova V., Alicino C., Almyroudi M.-P., Atchade E. (2019). Incidence and outcome of invasive candidiasis in intensive care units (ICUs) in Europe: Results of the EUCANDICU project. Crit. Care.

[2] Doi A.M., Pignatari A.C.C., Edmond M.B., Marra A.R., Camargo L.F.A., Siqueira R.A., da Mota V.P., Colombo A.L. (2016). Epidemiology and microbiologic characterization of nosocomial candidemia from a Brazilian national surveillance program. PLoS ONE.

[3] Mermel L.A., Allon M., Bouza E., Craven D.E., Flynn P., O’Grady N.P., Raad I.I., Rijnders B.J.A., Sherertz R.J., Warren D.K. (2009). Clinical practice guidelines for the diagnosis and management of intravascular catheter-related infection: 2009 update by the Infectious Diseases Society of America. Clin. Infect. Dis..

[4] Ben-Ami R., Weinberger M., Orni-Wasserlauff R., Schwartz D., Itzhaki A., Lazarovitch T., Bash E., Aharoni Y., Moroz I., Giladi M. (2008). Time to blood culture positivity as a marker for catheter-related candidemia. J. Clin. Microbiol..

[5] Pappas P.G., Kauffman C.A., Andes D.R., Clancy C.J., Marr K.A., Ostrosky-Zeichner L., Reboli A.C., Schuster M.G., Vazquez J.A., Walsh T.J. (2016). Clinical practice guideline for the management of candidiasis: 2016 update by the Infectious Diseases Society of America. Clin. Infect. Dis..

[6] Kuhn D.M., George T., Chandra J., Mukherjee P.K., Ghannoum M.A. (2002). Antifungal susceptibility of Candida biofilms: Unique efficacy of amphotericin B lipid formulations and echinocandins. Antimicrob. Agents Chemother..

[7] Uppuluri P., Chaturvedi A.K., Srinivasan A., Banerjee M., Ramasubramaniam A.K., Köhler J.R., Kadosh D., Lopez-Ribot J.L. (2010). Dispersion as an important step in the Candida albicans biofilm developmental cycle. PLoS Pathog..

[8] Rajendran R., Sherry L., Nile C.J., Sherriff A., Johnson E.M., Hanson M.F., Williams C., Munro C.A., Jones B.J., Ramage G. (2016). Biofilm formation is a risk factor for mortality in patients with Candida albicans bloodstream infection—Scotland, 2012-2013. Clin. Microbiol. Infect..

[9] Kullberg B.J., Arendrup M.C. (2015). Invasive candidiasis. N. Engl. J. Med..

[10] Morrell M., Fraser V.J., Kollef M.H. (2005). Delaying the empiric treatment of Candida bloodstream infection until positive blood culture results are obtained: A potential risk factor for hospital mortality. Antimicrob. Agents Chemother..

[11] Kollef M., Micek S., Hampton N., Doherty J.A., Kumar A. (2012). Septic shock attributed to Candida infection: Importance of empiric therapy and source control. Clin. Infect. Dis..

[12] Kobayashi T., Nakamura I., Machida M., Watanabe H. (2024). Clinical features of Candida catheter-related bloodstream infections and persistent infections associated with early catheter reinsertion: A 6-year retrospective study. J. Glob. Infect. Dis..

[13] Centers for Disease Control and Prevention (2024). Antifungal Susceptibility Testing for C. auris.

[14] Arendrup M.C., Guinea J., Arikan-Akdagli S., Meijer E.F., Meis J.F., Buil J.B., Dannaoui E., Giske C.G., Lyskova P., Meletiadis J. (2026). How to interpret MICs of amphotericin B, echinocandins and flucytosine against Candida auris (*Candidozyma auris*) according to the newly established European Committee for Antimicrobial Susceptibility Testing (EUCAST) breakpoints. Clin. Microbiol. Infect..

[15] Charlson M.E., Pompei P., Ales K.L., MacKenzie C.R. (1987). A new method of classifying prognostic comorbidity in longitudinal studies: Development and validation. J. Chronic Dis..

[16] Tiseo G., Vena A., Bassetti M., Bartalucci C., Cerchiaro M., Cesaretti M., Marchese A., Di Pilato V., Escribano P., Forniti A. (2025). Persistent candidemia caused by different Candida species: Data from a multicenter contemporary cohort. J. Infect..

[17] Guzman J.A., Tchokonte R., Sobel J.D. (2011). Septic shock due to candidemia: Outcomes and predictors of shock development. J. Clin. Med. Res..

[18] Tunay B., Aydin S. (2022). Investigation of inflammation-related parameters in patients with candidemia hospitalized in intensive care: A retrospective cohort study. Sci. Prog..

[19] Schupp T., Weidner K., Rusnak J., Jawhar S., Forner J., Dulatahu F., Brück L., Dudda J., Hoffmann U., Abba M. (2023). The Neutrophil-to-Lymphocyte-Ratio as Diagnostic and Prognostic Tool in Sepsis and Septic Shock. Clin. Lab..

[20] Wu H., Cao T., Ji T., Luo Y., Huang J., Ma K. (2024). Predictive value of the neutrophil-to-lymphocyte ratio in the prognosis and risk of death for adult sepsis patients: A meta-analysis. Front. Immunol..

[21] Pfaller M.A., Diekema D.J., Turnidge J.D., Castanheira M., Jones R.N. (2019). Twenty years of the SENTRY Antifungal Surveillance Program: Results for Candida species from 1997–2016. Open Forum Infect. Dis..

[22] Satoh K., Makimura K., Hasumi Y., Nishiyama Y., Uchida K., Yamaguchi H. (2009). *Candida auris* sp. nov., a novel ascomycetous yeast isolated from the external ear canal of an inpatient in a Japanese hospital. Microbiol. Immunol..

[23] Lockhart S.R., Etienne K.A., Vallabhaneni S., Farooqi J., Chowdhary A., Govender N.P., Colombo A.L., Calvo B., Cuomo C.A., Desjardins C.A. (2017). Simultaneous emergence of multidrug-resistant Candida auris on 3 continents confirmed by whole-genome sequencing and epidemiological analyses. Clin. Infect. Dis..

[24] Erdem H., Şakir-Yildirim S., Ankarali H., Batirel A., Kul G., Fidan G., El-Kholy A., Pekok A.U., Taher O.M.E.A., Bozkurt F. (2025). Managing Candida auris fungemias: The results of a prospective and international study. Antimicrob. Agents Chemother..

[25] EUCAST (2023). EUCAST Definitive Document E.Def 7.4. Method for the Determination of Broth Dilution Minimum Inhibitory Concentrations of Antifungal Agents for Yeasts.

[26] Arendrup M.C., Prakash A., Meletiadis J., Sharma C., Chowdhary A. (2017). Comparison of EUCAST and CLSI reference microdilution MICs of eight antifungal compounds for Candida auris and associated tentative epidemiological cutoff values. Antimicrob. Agents Chemother..

[27] Satlin M.J., Chen L., Patel G., Gomez-Simmonds A., Weston G., Kim A.C., Seo S.K., Rosenthal M.E., Sperber S.J., Jenkins S.G. (2017). Multicenter clinical and molecular epidemiological analysis of bacteremia due to carbapenem-resistant Enterobacteriaceae (CRE) in the CRE epicenter of the United States. Antimicrob. Agents Chemother..

[28] Pitiriga V., Campos E., Bakalis J., Sagris K., Georgiadis G., Saroglou G., Tsakris A. (2025). Effect of catheter dwell time on the risk of central line-associated bloodstream infections in central venous catheters and peripherally inserted central catheters. Antimicrob. Resist. Infect. Control.

[29] López-Cortés L., Almirante B., Cuenca-Estrella M., Garnacho-Montero J., Padilla B., Puig-Asensio M., Ruiz-Camps I., Rodríguez-Baño J., Muñoz P., Guinea J. (2016). Empirical and targeted therapy of candidemia with fluconazole versus echinocandins: A propensity score-derived analysis of a population-based, multicentre prospective cohort. Clin. Microbiol. Infect..

